# Supplementary material for: Trends and projections of under-5 mortality in Bangladesh including the effects of maternal high-risk fertility behaviours and use of healthcare services
Source: PLoS One. 2021 Feb 4;16(2):e0246210. doi: 10.1371/journal.pone.0246210 (PMC7861360; doi:10.1371/journal.pone.0246210)
Supplement: S1 Table — (DOCX) [file pone.0246210.s001.docx]

**Supplementary Tables**

**S1 Table.** Information on Model Fittings of linear Regression Model.

| **Models** | **Measures** | **Constant (a_0_)** | **Coefficient (a_1_)** | **Robust standard**  **error (e)** | **χ^2^** | **ρ_cv_^2^** | **p-value** |
| --- | --- | --- | --- | --- | --- | --- | --- |
| 1 | Neonatal mortality | 2088.081 | -1.023 | 0.031 | 1663.810 | 0.994 | <0.001 |
| 2 | Infant mortality | 3937.399 | -1.933 | 0.238 | 529.750 | 0.896 | <0.001 |
| 3 | Under-5 mortality | 4286.435 | -2.103 | 0.279 | 751.760 | 0.937 | <0.001 |
|  | **Under-5 mortality by Socio-demographic characteristics** | | | |  |  |  |
|  | **Residence** |  |  |  |  |  |  |
| 4 | Urban | 2012.654 | -0.984 | 0.754 | 599.15 | 0.990 | <0.001 |
| 5 | Rural | 3693.132 | -1.809 | 0.349 | 477.970 | 0.942 | <0.001 |
|  | **Economic status** |  |  |  |  |  |  |
| 6 | Poor | 5681.228 | -2.789 | 0.541 | 54.800 | 0.886 | <0.001 |
| 7 | Middle | 8091.843 | -3.981 | 0.992 | 218.570 | 0.978 | <0.001 |
| 8 | Rich | 5851.512 | -2.881 | 0.291 | 109.580 | 0.996 | <0.001 |

**Note:** As $\rho_{cv}^{2}$ is tending to be 1 indicates that the models are a better fit.
