# Supplementary material for: Trends and projections of under-5 mortality in Bangladesh including the effects of maternal high-risk fertility behaviours and use of healthcare services
Source: PLoS One. 2021 Feb 4;16(2):e0246210. doi: 10.1371/journal.pone.0246210 (PMC7861360; doi:10.1371/journal.pone.0246210)
Supplement: S2 Table — (DOCX) [file pone.0246210.s002.docx]

**S2 Table.** Information about plotting of Fig 1 and Fig 2.

| **Measures** | **Frequency (%) in Year** | | | | | | |
| --- | --- | --- | --- | --- | --- | --- | --- |
|  | **1994** | **1997** | **2000** | **2004** | **2007** | **2011** | **2014** |
| **Maternal high-risk fertility behaviors** | | | | | | | |
| Age at birth <18 years | 16.9 | 15.4 | 14.8 | 17.5 | 18.2 | 16.0 | 13.3 |
| Age at birth >34 years | 7.0 | 9.5 | 9.5 | 6.8 | 6.0 | 5.3 | 4.3 |
| Birth interval <24 months | 16.1 | 18.4 | 17.6 | 16.4 | 15.1 | 11.8 | 11.7 |
| Birth order >3 | 35.4 | 43.7 | 40.0 | 27.6 | 23.6 | 18.3 | 15.2 |
| Multiple risk | 17.4 | 22.9 | 21.0 | 16.2 | 15.1 | 11.1 | 7.3 |
| **Healthcare service utilization** | | | | | | | |
| Taken ANC at least 4 times | 5.4 | 6.9 | 10.5 | 15.9 | 20.6 | 26.4 | 31.8 |
| Institutional delivery | 3.8 | 5.7 | 9.2 | 10.1 | 15.0 | 27.4 | 37.9 |
| Cesarean section delivery | - | - | 3.0 | 3.7 | 7.9 | 15.4 | 23.9 |
| Delivery by skilled birth attendant | 13.4 | 15.6 | 21.8 | 13.4 | 27.7 | 30.8 | 52.9 |

**Note:** The sample were weighted.
