# Supplementary material for: Trends and projections of under-5 mortality in Bangladesh including the effects of maternal high-risk fertility behaviours and use of healthcare services
Source: PLoS One. 2021 Feb 4;16(2):e0246210. doi: 10.1371/journal.pone.0246210 (PMC7861360; doi:10.1371/journal.pone.0246210)
Supplement: Attachment — Submitted filename: Rebuttal Letter.docx [file pone.0246210.s003.docx]

**Rebuttal Letter**

September 23, 2020

Petra Baltigh
Handling Academic Editor
PLOS ONE

**Re: Previous Manuscript Number PONE-D-20-09101**

Dear Editor,

Thank you very much for your kind consideration of our manuscript, previously entitled, ***“***Trends in, and projections of under-5 mortality in Bangladesh: are maternal risk factors enhancing under-5 mortality risk?***”*** We have revised the manuscript according to the comments by the editors.

*Current manuscript submitted under manuscript no. PONE-D-20-29506, entitled, “Trends in, and projections of under-5 mortality (U5M) in Bangladesh: effects of maternal high-risk fertility behaviors and healthcare service utilization on U5M.”*

We hope that the revisions are satisfactory in addressing previous issues raised by the editor, and look forward to hearing your decision about this article.

Regards

Md. Rafiqul Islam, PhD

Professor, Department of Population Science and Human Resource Development,

University of Rajshahi, Rajshahi-6205, Bangladesh.

E-mail: [rafique_pops@yahoo.com](mailto:rafique_pops@yahoo.com)

**Editors comment to address**

- Our editorial staff has assessed your submission, and we have significant concerns about the grammar, usage, and overall readability of the manuscript. We believe that the manuscript is much more likely to be accepted if it is easy for the reviewers to read and understand. We therefore request that you revise the text to fix the grammatical errors and improve the overall readability of the text before we send it for review. We suggest you have a fluent, preferably native, English-language speaker thoroughly copyedit your manuscript for language usage, spelling, and grammar. If you do not know anyone who can do this, you may wish to consider employing a professional scientific editing service.

**Response:** We have revised our current manuscript submitted under manuscript no. PONE-D-20-29506, entitled, “Trends in, and projections of under-5 mortality (U5M) in Bangladesh: effects of maternal high-risk fertility behaviors and healthcare service utilization on U5M.” We have improved the grammar, usage, and overall readability of the manuscript and the manuscript is revised by -

- Md. Nuruzzaman Khan, PhD fellow, School of Public Health and Medicine, Faculty of Health and Medicine, The University of Newcastle, Callaghan NSW 2308, Australia
- Dr. Hafiz TA Khan, Professor of Public Health & Statistics, College of Nursing, Midwifery and Healthcare, University of West London, Paragon House, Boston Manor Road, Brentford TW8 9GB, United Kingdom.

We are, therefore, hoping for your positive response.
