# Supplementary material for: Trends and projections of under-5 mortality in Bangladesh including the effects of maternal high-risk fertility behaviours and use of healthcare services
Source: PLoS One. 2021 Feb 4;16(2):e0246210. doi: 10.1371/journal.pone.0246210 (PMC7861360; doi:10.1371/journal.pone.0246210)
Supplement: Attachment — Submitted filename: Response to Reviewers.docx [file pone.0246210.s004.docx]

November 25, 2020

Russell Kabir, PhD
Academic Editor
PLOS ONE

**Re:** Revised manuscript submission: **MS ID# PONE-D-20-29506**

Dear Dr Kabir,

Thank you for giving us this opportunity of submitting a revised version of our manuscript: *Trends and projections of under-5 mortality in Bangladesh including the effects of maternal high-risk fertility behaviours and use of healthcare services.* We found the reviewers’ comments very useful and they have allowed us to improve the paper.

**As per your suggestion, we have now submitted the following documents:**

- A rebuttal letter that responds to comments raised by the reviewers. This letter has been uploaded as a separate file and labelled **'Response to Reviewers'.**
- A marked-up copy of our manuscript that highlights changes made to the original version. This has been uploaded as a separate file and labelled '**Revised Manuscript with Track Changes'**.
- An unmarked version of the revised paper without tracked changes. This has also been uploaded as a separate file and labelled **'Revised Manuscript'**.

We would like to inform you that as per your advice one of our colleague, who is native in English (not listed in the author’s list) edited the manuscript to correct the grammatical errors, and improve readability, clarity and quality of the manuscripts. Formatting issues raised by the journal’s office have addressed as per the journal guidelines. We hope you will find our revised version acceptable.

Yours sincerely,

Md. Rafiqul Islam, PhD (on the behalf of co-authors)

Professor

Department of Population Science and Human Resource Development

University of Rajshahi

Rajshahi-6205, Bangladesh.

**Journal Requirements:**

1. Please ensure that your manuscript meets PLOS ONE's style requirements, including those for file naming. The PLOS ONE style templates can be found at

<https://journals.plos.org/plosone/s/file?id=wjVg/PLOSOne_formatting_sample_main_body.pdf>

<https://journals.plos.org/plosone/s/file?id=ba62/PLOSOne_formatting_sample_title_authors_affiliations.pdf>

**Response**: We have revised the manuscript as per PLOS ONE’s guidelines.

2.We suggest you thoroughly copyedit your manuscript for language usage, spelling, and grammar. If you do not know anyone who can help you do this, you may wish to consider employing a professional scientific editing service.  

Whilst you may use any professional scientific editing service of your choice, PLOS has partnered with both American Journal Experts (AJE) and Editage to provide discounted services to PLOS authors. Both organizations have experience helping authors meet PLOS guidelines and can provide language editing, translation, manuscript formatting, and figure formatting to ensure your manuscript meets our submission guidelines. To take advantage of our partnership with AJE, visit the AJE website (http://learn.aje.com/plos/) for a 15% discount off AJE services. To take advantage of our partnership with Editage, visit the Editage website ([www.editage.com](http://www.editage.com/)) and enter referral code PLOSEDIT for a 15% discount off Editage services.  If the PLOS editorial team finds any language issues in text that either AJE or Editage has edited, the service provider will re-edit the text for free.

 Upon resubmission, please provide the following:

- The name of the colleague or the details of the professional service that edited your manuscript
- A copy of your manuscript showing your changes by either highlighting them or using track changes (uploaded as a *supporting information* file)
- A clean copy of the edited manuscript (uploaded as the new *manuscript* file)

**Response:** As per your advice one of our colleague, who is native in English (Dr Helen Findlay, not listed in the author’ list) edited the manuscript to correct grammar, and improve readability, clarity and quality of the manuscript.

3. Please consider modifying your title to ensure that it is specific, descriptive, concise, and comprehensible to readers outside the field (for example by not using acronyms in the title).

**Response:** We have modified the title.

4. In your Data Availability statement, you have not specified where the minimal data set underlying the results described in your manuscript can be found. PLOS defines a study's minimal data set as the underlying data used to reach the conclusions drawn in the manuscript and any additional data required to replicate the reported study findings in their entirety. All PLOS journals require that the minimal data set be made fully available. For more information about our data policy, please see <http://journals.plos.org/plosone/s/data-availability>.

Upon re-submitting your revised manuscript, please upload your study’s minimal underlying data set as either Supporting Information files or to a stable, public repository and include the relevant URLs, DOIs, or accession numbers within your revised cover letter. For a list of acceptable repositories, please see <http://journals.plos.org/plosone/s/data-availability#loc-recommended-repositories>. Any potentially identifying patient information must be fully anonymized.

Important: If there are ethical or legal restrictions to sharing your data publicly, please explain these restrictions in detail. Please see our guidelines for more information on what we consider unacceptable restrictions to publicly sharing data: <http://journals.plos.org/plosone/s/data-availability#loc-unacceptable-data-access-restrictions>. Note that it is not acceptable for the authors to be the sole named individuals responsible for ensuring data access.

We will update your Data Availability statement to reflect the information you provide in your cover letter.

**Response:** The data underlying this study (BDHSs, 1993-2014) are third party data, and are available from the MEASURE DHS Archive via the instructions included in the following link: http://dhsprogram.com/data/Using-Datasets-for-Analysis.cfm.

5. Please amend your list of authors on the manuscript to ensure that each author is linked to an affiliation. Authors’ affiliations should reflect the institution where the work was done (if authors moved subsequently, you can also list the new affiliation stating “current affiliation:….” as necessary).

**Response:** The affiliations are mentioned accordingly.

6. Please upload a copy of Figure 2, to which you refer in your text on page 26. If the figure is no longer to be included as part of the submission please remove all reference to it within the text.

**Response:** Uploaded the figure accordingly.

7. Please include captions for your Supporting Information files at the end of your manuscript, and update any in-text citations to match accordingly. Please see our Supporting Information guidelines for more information: http://journals.plos.org/plosone/s/supporting-information.

**Response:** Included accordingly.

8. Thank you for stating the following in the Competing Interests section:

"The authors have declared that no competing interests exist."

We note that one or more of the authors are employed by a commercial company: MEL and Research.

**Response:** None of the authors have any competing interest in current study. The affiliation of MEL and Research, Practical Action do not have any role in this study.

8.1. Please provide an amended Funding Statement declaring this commercial affiliation, as well as a statement regarding the Role of Funders in your study. If the funding organization did not play a role in the study design, data collection and analysis, decision to publish, or preparation of the manuscript and only provided financial support in the form of authors' salaries and/or research materials, please review your statements relating to the author contributions, and ensure you have specifically and accurately indicated the role(s) that these authors had in your study. You can update author roles in the Author Contributions section of the online submission form.

**Response:** There was no funding source for this study or the affiliation of MEL and Research, Practical Action do not have any role in this study.

Please also include the following statement within your amended Funding Statement.

“The funder provided support in the form of salaries for authors [insert relevant initials], but did not have any additional role in the study design, data collection and analysis, decision to publish, or preparation of the manuscript. The specific roles of these authors are articulated in the ‘author contributions’ section.”

If your commercial affiliation did play a role in your study, please state and explain this role within your updated Funding Statement.

**Response:** There was no funding source for this study or the affiliation of MEL and Research, Practical Action do not have any role in this study.

8.2. Please also provide an updated Competing Interests Statement declaring this commercial affiliation along with any other relevant declarations relating to employment, consultancy, patents, products in development, or marketed products, etc.

Within your Competing Interests Statement, please confirm that this commercial affiliation does not alter your adherence to all PLOS ONE policies on sharing data and materials by including the following statement: "This does not alter our adherence to PLOS ONE policies on sharing data and materials.” (as detailed online in our guide for authors http://journals.plos.org/plosone/s/competing-interests). If this adherence statement is not accurate and there are restrictions on sharing of data and/or materials, please state these. Please note that we cannot proceed with consideration of your article until this information has been declared.

Please include both an updated Funding Statement and Competing Interests Statement in your cover letter. We will change the online submission form on your behalf.

Please know it is PLOS ONE policy for corresponding authors to declare, on behalf of all authors, all potential competing interests for the purposes of transparency. PLOS defines a competing interest as anything that interferes with, or could reasonably be perceived as interfering with, the full and objective presentation, peer review, editorial decision-making, or publication of research or non-research articles submitted to one of the journals. Competing interests can be financial or non-financial, professional, or personal. Competing interests can arise in relationship to an organization or another person. Please follow this link to our website for more details on competing interests: http://journals.plos.org/plosone/s/competing-interests

**Response:** The authors received no specific funding for this work, neither have any competing interest. There was no funding source for this study or the affiliation of MEL and Research, Practical Action do not have any role in this study.

**Reviewer #1**

While the statistical analysis is comprehensive, the manuscript requires extensive language editing- which affects the overall readability of the manuscript.

As an example: line 78 '.... South Asian countries that placing it in the 3rd 78 positioned country after Pakistan' should probably read'.... South Asian countries which places it in the third position after Pakistan'. There are several other instances of incomplete and ungrammatical sentences, for example line 79, 90, 92, 140-142 all in the introduction require language editing.

Other than that, the manuscript is sound.

**Response:** Thank you for your appreciations and suggestions. As per your advice one of our colleague, who is native in English (Dr Helen Findlay, not listed in the author’ list) edited the manuscript to correct grammar, and improve readability, clarity and quality of the contents.

**Reviewer #2:**

In the present work entitled “Trends in, and projections of under-5 mortality (U5M) in Bangladesh: effects of maternal high-risk fertility behaviors and healthcare service utilization on U5M”, authors presented important aspects with sound analytical explanations for the policymakers. Here, the authors projected the under-5 mortality (U5M) in Bangladesh and explored its associations with maternal high-risk fertility behaviors and utilization of available healthcare services. The authors presented a very sound analytical piece of work, and then justified its significance using the real-life application.

**Response:** Thank you for your appreciations

I have a major concern, which needs to be addressed in the manuscript.

In line no. 152, authors used the information obtained from the women that “Did you give birth to any children within five years”. In the situations of twins and multiple births within 5 years, how the additional information “Is the child alive?” addresses the living status of children.

Authors should elaborate their explanations of outcome variables for a more practical situation and also how the model incorporated those aspects.

**Response:** The respondents were asked follow-up questions for every child if they have given birth to multiple children within 5 years prior to the survey date. All the predictors are taken in the model was based on existing global literature.
